# Genome-wide analysis of *Cyclophilin* gene family in soybean (*Glycine max*)

**DOI:** 10.1186/s12870-014-0282-7

**Published:** 2014-10-29

**Authors:** Hemanta Raj Mainali, Patrick Chapman, Sangeeta Dhaubhadel

**Affiliations:** Department of Biology, University of Western Ontario, London, ON Canada; Agriculture and Agri-Food Canada, 1391 Sandford Street, London, ON Canada

## Abstract

**Background:**

Cyclophilins (CYPs) belong to the immunophilin superfamily, and have peptidyl-prolyl *cis-trans* isomerase (PPIase) activity. PPIase catalyzes *cis-* and *trans-*rotamer interconversion of the peptidyl-prolyl amide bond of peptides, a rate-limiting step in protein folding. Studies have demonstrated the importance of many PPIases in plant biology, but no genome-wide analysis of the *CYP* gene family has been conducted for a legume species.

**Results:**

Here we performed a comprehensive database survey and identified a total of 62 *CYP* genes, located on 18 different chromosomes in the soybean genome (*GmCYP1* to *GmCYP62*), of which 10 are multi- and 52 are single-domain proteins. Most of the predicted GmCYPs clustered together in pairs, reflecting the ancient genome duplication event. Analysis of gene structure revealed the presence of introns in protein-coding regions as well as in 5′ and 3′ untranslated regions, and that their size, abundance and distribution varied within the gene family. Expression analysis of *GmCYP* genes in soybean tissues displayed their differential tissue specific expression patterns.

**Conclusions:**

Overall, we have identified 62 *CYP* genes in the soybean genome, the largest *CYP* gene family known to date. This is the first genome-wide study of the *CYP* gene family of a legume species. The expansion of *GmCYP* genes in soybean, and their distribution pattern on the chromosomes strongly suggest genome-wide segmental and tandem duplications.

**Electronic supplementary material:**

The online version of this article (doi:10.1186/s12870-014-0282-7) contains supplementary material, which is available to authorized users.

## Background

Cyclophilins (CYPs) are ubiquitous proteins found in organisms ranging from archaea and bacteria to plants and animals [1,2]. As they were originally identified as receptors for the immunosuppressive drug cyclosporine A (CsA), CYPs are classified in the immunophilin family of proteins possessing peptidyl prolyl *cis/trans* isomerase activity. Multiple CYPs have been found in genomes of various prokaryotes, but only a few have been studied in detail. The *Escherichia coli* genome encodes two CYPs, a cytosolic form (EcCYP-18) and its periplasmic counterpart (EcCYP-20) [3]. In the yeast *Saccharomyces cerevisiae* there are at least 8 different CYPs, *Cpr1* to *Cpr8* [4]. These proteins are not essential for growth, but are crucial for survival after heat stress [5]. The human genome encodes 16 unique CYPs, categorized into 7 major groups, namely human CYP A (hCYP-A), hCYP-B, hCYP-C, hCYP-D, hCYP-E, hCYP-40 and hCYP-NK [6]. The hCYP-A binds to CsA, and forms a ternary complex with calcineurin. The CsA-hCYP-A binding to calcineurin inhibits the phosphatase activity of calcineurin that results in a cascade of activities leading to the inactivation of T-cells [7].

Compared to human CYPs, very little is known about plant CYPs. The first plant *CYPs* were identified concurrently from tomato (*Lycopersicon esculentum*), maize (*Zea mays*), and oilseed rape (*Brassica napus*) [8]. Recently, with the availability of whole genome sequencing, the identification and characterization of plant CYPs has progressed substantially. However, compared to other organisms, the total number of plant *CYPs* in databases is still small, which suggests that many plant *CYPs* remain to be identified [9]. To date, *Arabidopsis thaliana* and rice (*Oryza sativa*) are the two plant species reported to have highest number of CYPs with 35 *AtCYPs* [10,11] and 28 *OsCYPs* [10,12], respectively. Among the identified *AtCYPs*, only 15 are characterized at the molecular level [11,13–21]. Their encoded proteins are found in the cytoplasm [17,19,20], endoplasmic reticulum (ER) [18,21], chloroplast [15,16], and nucleus [13]. An increase in the expression of *ROC1,* an *AtCYP,* in response to light is associated with phytochromes and cryptochromes [19,22]. *roc1* mutants display an early flowering phenotype [22], while gain-of-function mutations in *ROC1* reduce stem elongation and increase shoot branching [23]. In contrast, loss-of-function mutations in *AtCYP40* reduce the number of juvenile leaves, with no change in inflorescence morphology or flowering time, and *Arabidopsis* plants with a defective *AtCYP20-3* are found to be hypersensitive to oxidative stress conditions created by high light and high salt levels, and osmotic shock [24]. In addition to the AtCYPs having roles in various developmental processes, AtCYP59, a multi-domain CYP with a RNA recognition motif (RRM), regulates transcription and pre-mRNA processing by binding to the C-terminal domain of RNA polymerase II [13]. Collectively, these results show the roles of *Arabidopsis* CYPs in different cellular pathways, which necessitate further work to explore the function associated with each of the CYPs.

Compared to the *Arabidopsis* CYPs, little work has been done on the rice CYPs. Most of the studies on the latter show their roles in different types of stresses. *OsCYP2* has been reported to have a role in different abiotic stress responses [25]. The expression of *OsCYP2* is up-regulated towards salt stress, and its over-expression in rice enhances tolerance towards the salt stress. Similarly, overexpression in *Arabidopsis* and tobacco of the thylakoid-localized *OsCYP20-2* increased tolerance towards osmotic stress, and to extremely high light conditions [26]. The expression levels of several other *OsCYPs* were increased by abiotic stresses such as desiccation and salt stress [10,12], indicating a critical role of OsCYPs during stress conditions.

Soybean (*Glycine max* [L.] Merr) is a legume plant belonging to the *Papilionoideae* family and is a rich source of protein, oil and plant natural products such as isoflavonoids. The soybean genome contains 56,044 protein coding loci located on 20 different chromosomes. Soybean has undergone two whole genome duplication events approximately 59 and 13 million years ago, as a result of which 75% of the genes have multiple copies [27]. Until now, not much was known about soybean CYPs except that a handful of *CYP* gene sequences had been deposited in the public databases. We present here a genome-wide identification of soybean *CYP*s, their phylogenetic analysis, chromosomal distribution, and structural and expressional analysis*.* Our results indicate that soybean contains 62 CYPs, the largest family of CYP known to date in any organism. Further, the study describes a genome-wide segmental and tandem duplication during expansion of the *GmCYP* gene family.

## Results and discussion

### The soybean genome contains 62 putative *GmCYPs*

**Figure 1:**
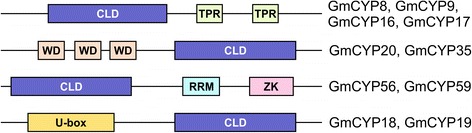
**Schematic representation of multi-domain GmCYPs.** CLD, cyclophilin-like-domain; TPR, tetratricopeptide repeat; WD, tryptophan-aspartate repeat; RRM, RNA recognition motif; ZK, zink knuckle, U-box, U-box domain.

To identify all the members of the *CYP* gene family in soybean, a BLASTN search of the soybean genome database *G. max* Wm82.a2.v1 (http://phytozome.jgi.doe.gov/pz/portal.html#!search?show=BLAST&method=Org_Gmax) was performed using the nucleotide sequence of a previously identified soybean *CYP* cDNA (GenBank: AF456323) as a query. This search identified 11 unique *CYP* genes. Each of the 11 *CYP* genes was used separately as a query sequence in the BLAST search of soybean genome database. This process was repeated until no new *CYP* gene was found. A total of 62 soybean *CYPs,* located on 18 different chromosomes, were identified and named *GmCYP1* to *GmCYP62* (Table 1). Of the 62 *GmCYPs*, 52 encoded a protein with a single cyclophilin-like domain (CLD) which is responsible for the *cis/trans* isomerization of the peptidyl prolyl peptide bond. The remaining 10 GmCYPs contained the CLD and additional domains. As shown in Figure 1, GmCYP8, GmCYP9, GmCYP16, and GmCYP17 each contained two tetratricopeptide repeats (TPRs) at the C-terminus. The TPR motif is degenerate in nature and consists of a 34 amino acid repeat unit typically arranged in tandem arrays [28]. Such TPR motif containing proteins mediate protein-protein interactions and often help in the assembly of multi-protein complexes. AtCYP40 (AGI:At2g15790), the *Arabidopsis* ortholog of GmCYP8, GmCYP9, GmCYP16, and GmCYP17, contains 3 TPRs and is involved in microRNA-mediated gene regulation [29]. Loss-of-function mutation of *AtCYP40* showed a precocious phase change with reduced number of juvenile leaves, but no alteration of flowering time [20]. Moreover, the conserved amino acids of the TPR domain of AtCYP40 are required for the interaction between AtCYP40 and cytoplasmic Hsp90 proteins. This interaction is essential for the function of AtCYP40 *in planta* [29] suggesting a critical role for the TPR domain in microRNA-mediated gene regulation. Here we speculate a possibly similar function for the TPR domain in GmCYP8, GmCYP9, GmCYP16 and/or GmCYP17.

**Table 1:** **Soybean** ***cyclophilin*** **gene family**

**Gene name**	**Predicted transcript size (bp)**	**Predicted protein size (AA)**	**Predicted subcellular location**	**Domain information**
*GmCYP1*	973	172	Cytosol	SD
*GmCYP32*	1595	337	Secretory	SD
*GmCYP2*	1224	172	Cytosol	SD
*GmCYP33*	1301	373	Secretory	SD
*GmCYP3*	854	172	Cytosol	SD
*GmCYP34*	1065	204	Secretory	SD
*GmCYP4*	775	172	Cytosol	SD
*GmCYP35*	2543	616	Cytosol	MD
*GmCYP5*	354	117	Cytosol	SD
*GmCYP36*	2559	668	Nucleus	SD
*GmCYP6*	393	130	Cytosol	SD
*GmCYP37*	1982	493	Cytosol	SD
*GmCYP7*	1072	175	Cytosol	SD
*GmCYP38*	582	114	Cytosol	SD
*GmCYP8*	1611	360	Cytosol	MD
*GmCYP39*	1233	232	Secretory	SD
*GmCYP9*	1241	360	Cytosol	MD
*GmCYP40*	1264	236	Secretory	SD
*GmCYP10*	1380	253	Chloroplast	SD
*GmCYP41*	1238	236	Secretory	SD
*GmCYP11*	1062	175	Cytosol	SD
*GmCYP42*	1087	165	Cytosol	SD
*GmCYP12*	711	236	Chloroplast	SD
*GmCYP43*	3138	850	Nucleus	SD
*GmCYP13*	793	164	Cytosol	SD
*GmCYP44*	2766	167	Secretory	SD
*GmCYP14*	1253	260	Chloroplast	SD
*GmCYP45*	1085	226	Secretory	SD
*GmCYP15*	1200	221	Cytosol	SD
*GmCYP46*	2532	843	Nucleus	SD
*GmCYP16*	1745	361	Cytosol	MD
*GmCYP47*	1836	387	Chloroplast	SD
*GmCYP17*	1770	361	Cytosol	MD
*GmCYP48*	1751	439	Chloroplast	SD
*GmCYP18*	2576	597	Nucleus	MD
*GmCYP49*	1324	225	Mitochondria	SD
*GmCYP19*	2292	597	Nucleus	MD
*GmCYP50*	2922	227	Chloroplast	SD
*GmCYP20*	2554	616	Nucleus	MD
*GmCYP51*	947	232	Mitochondria	SD
*GmCYP21*	967	194	Cytosol	SD
*GmCYP52*	1724	439	Chloroplast	SD
*GmCYP22*	947	183	Cytosol	SD
*GmCYP53*	1983	445	Chloroplast	SD
*GmCYP23*	1349	251	Chloroplast	SD
*GmCYP54*	3559	849	Nucleus	SD
*GmCYP24*	1118	204	Secretory	SD
*GmCYP55*	1175	286	Chloroplast	SD
*GmCYP25*	1061	235	Secretory	SD
*GmCYP56*	2537	633	Nucleus	MD
*GmCYP26*	1459	238	Secretory	SD
*GmCYP57*	546	181	Cytosol	SD
*GmCYP27*	2693	659	Nucleus	SD
*GmCYP58*	2374	350	Chloroplast	SD
*GmCYP28*	1869	263	Chloroplast	SD
*GmCYP59*	2403	640	Nucleus	MD
*GmCYP29*	1822	326	Secretory	SD
*GmCYP60*	1921	445	Chloroplast	SD
*GmCYP30*	1988	327	Secretory	SD
*GmCYP61*	1493	230	Mitochondria	SD
*GmCYP31*	1645	337	PM/Mitochondria#	SD
*GmCYP62*	1489	292	Mitochondria	SD

#, prediction with low confidence.

GmCYP20 and GmCYP35 contain three tryptophan-aspartate (WD) repeats at the N-terminus (Figure 1). WD repeat-containing proteins are involved in a wide variety of cellular functions, providing binding sites for two or more proteins, or fostering transient interactions with other proteins [30,31]. The *Arabidopsis* CYP, AtCYP71 (AGI:At3g44600), contains 2 WD repeats [11] and functions in chromatin remodelling [14]. The very high sequence identity of AtCYP71 with GmCYP20 (87%) and GmCYP35 (83%) suggests that these two GmCYPs may play similar roles in soybean.

The sequence analysis identified two soybean CYPs, GmCYP56 and GmCYP59, having an RNA recognition motif (RRM) and zinc knuckle (ZK) at the C-terminus along with CLD at the N-terminus end (Figure 1). RRM is a small RNA binding motif of 90 amino acids and is conserved in a wide variety of organisms [32]. AtCYP59 (AGI:At1g53720), the *Arabidopsis* ortholog of GmCYP56 (80%) and GmCYP59 (66%) (Additional file 1) [11], contains an RRM motif, and is a transcriptional regulator [13] that interacts with the conserved sequence of unprocessed mRNA, leading to the inhibition of the PPIase activity *in vitro* [33]. Based on the functional association of AtCYP59 in transcriptional regulation, we speculate that the multi-domain soybean CYPs GmCYP56 and GmCYP59 possibly play a role in regulation of transcription in soybean *via* their RRMs.

Lastly, GmCYP18 and GmCYP19 contain a U-box at the N-terminus end of the protein. The U-box domain is highly conserved in some ubiquitin ligases and predicted to be a part of the ubiquitination machinery. Mammalian CYC4 and *Arabidopsis* AtCYP65 are the U-box containing CYP where the CYP domain is predicted to have chaperone function [11,34].

Of the 62 *GmCYPs*, it was ascertained that 13 contain a chloroplast transit peptide, 13 contain a signal peptide, 5 contain a mitochondrial targeting peptide, 10 contain a nuclear localization signal, and the remaining 21 are cytosolic (Table 1). Unlike *Arabidopsis* and rice CYPs [10], none of the soybean CYPs are predicted to be localized to the ER or golgi or plasma membrane. Only one secretory GmCYP, GmCYP39, is predicted for localization in the mitochondrial inner membrane or plasma membrane. A search for ER retention signal did not locate KDEL or HDEL in any GmCYP. We also searched for *CYP* genes in the DFCI soybean gene index that contains 1,354,268 ESTs representing 73,178 TC sequences (http://compbio.dfci.harvard.edu/tgi/). Screening this database confirmed that 15 of the 62 *GmCYP* genes we identified were represented with 99-100% identity, and 100% coverage, implying that at least 25% of the *GmCYPs* are transcribed in various soybean tissues during normal growth and development, or in response to stress. Additionally, 33 *GmCYPs* displayed greater than 95% sequence identity with TC sequences in the soybean EST database, but with less than 100% query coverage. The lower sequence identities could be due simply to cultivar-specific sequence differences between the two databases, with the whole genome sequence originating solely from the cultivar Williams82 [27], and the DFCI soybean gene index comprising EST data from cDNA libraries of several different soybean cultivars, the number of transcribed *GmCYPs* in soybean can be expected to be more than 15. A list of all the soybean *CYP* gene family members and their detailed information is provided in Additional file 1.

### Chromosomal distribution and phylogenetic analysis of soybean *CYP* genes

**Figure 2:**
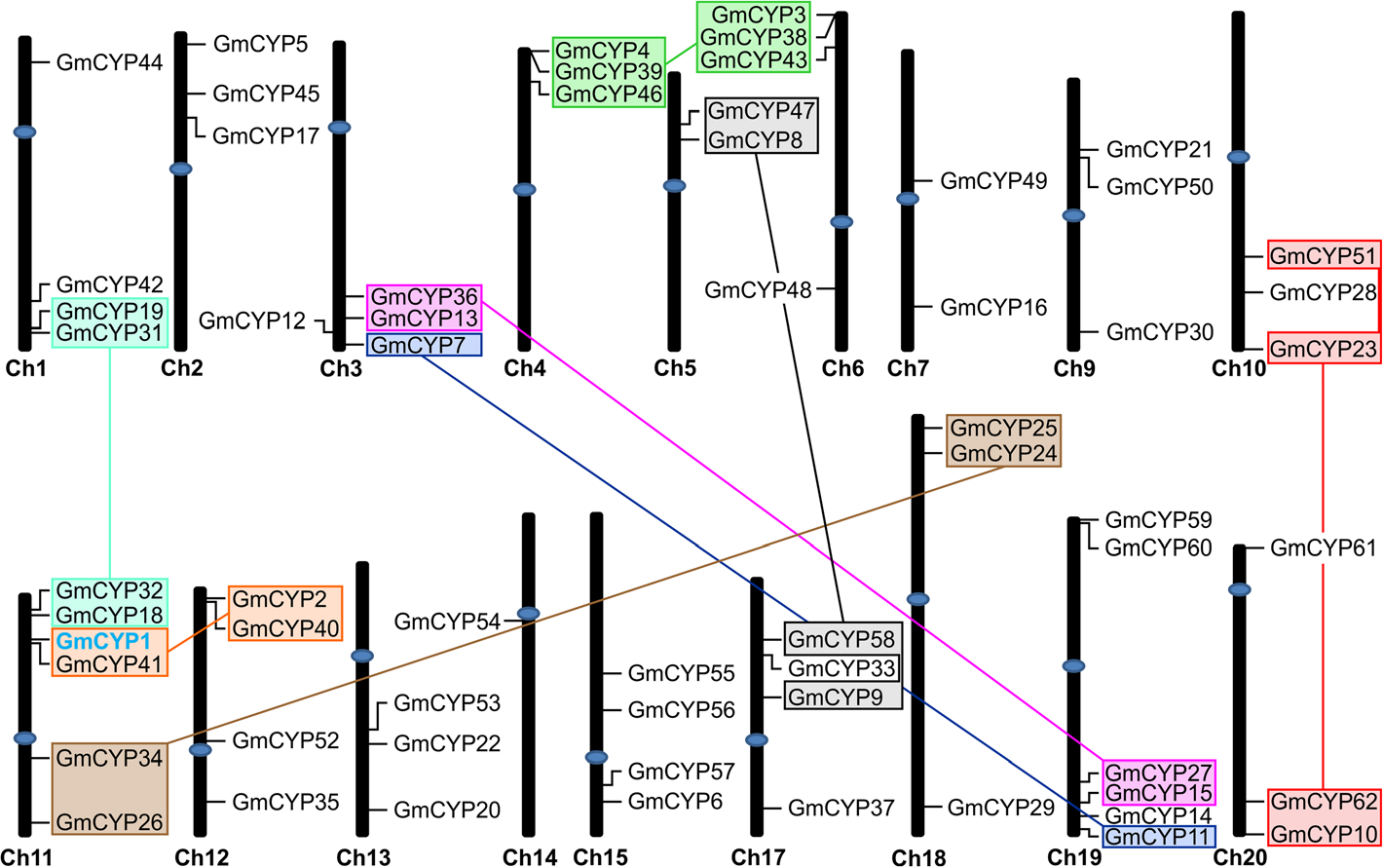
**Genomic distributions of** ***GmCYP*** **genes on soybean chromosomes** ***.*** Chromosomal locations of *GmCYPs* are indicated based on the location of the genes, length of chromosomes and positions of centromeres. The chromosomes are drawn to scale and chromosome numbers are shown under each chromosome. The *GmCYPs* that are clustered together and speculated to have undergone segmental duplication are indicated by shaded boxes of the same color and connected to each other by a line. Centromeres are indicated by blue ovals.

To determine the genome organization and distribution of *GmCYPs* on different chromosomes in soybean, a chromosome map was constructed. The results showed that the 62 *GmCYPs* are located on 18 different chromosomes. As depicted in Figure 2, the gene density per chromosome is uneven. Chromosome 11 and 19 contain the most, and show a relatively dense occurrence of *CYP* genes (6 each), whereas only one *CYP* (*GmCYP54*) is present on chromosome 14. No *CYPs* were found on chromosome 8 or 16. Most *GmCYPs* were localized towards the chromosome ends, and only *GmCYP52, GmCYP49* and *GmCYP54* were found near centromeres (Figure 2), suggesting the possibility of inter-chromosomal rearrangements, after genome duplication, between different soybean chromosomes.

**Figure 3:**
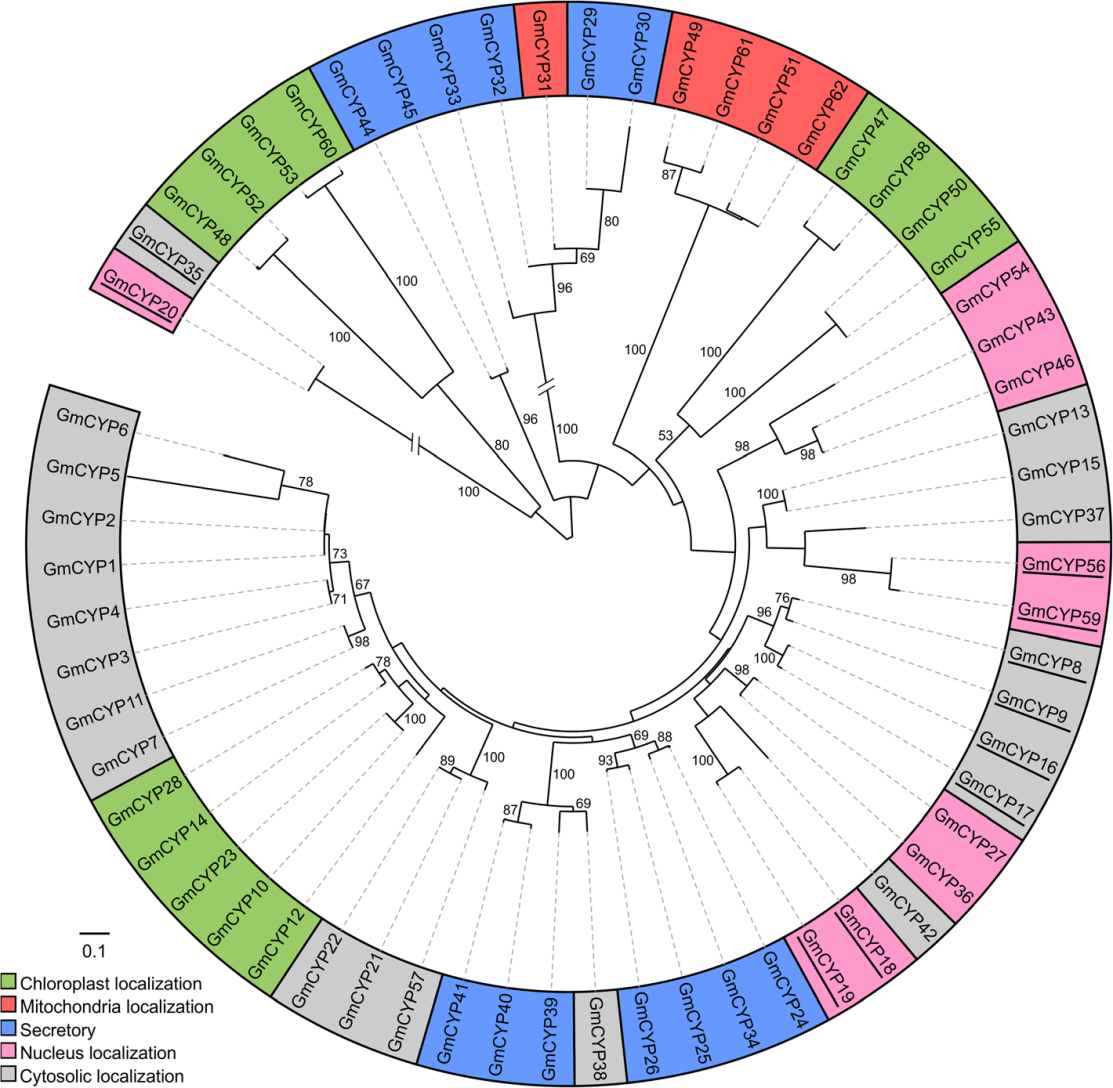
**Phylogenetic relationships of GmCYP proteins.** A Neighbor-Joining tree was generated by MEGA5.1 software [51] using putative amino acid sequence of 62 GmCYPs, and the tree was annotated using Interactive Tree of Life [52]. The numbers next to the branch shows the 1000 bootstrap replicates expressed in percentage. The solid line represents the real branch length and dotted lines added later for better visualization. The multi-domain CYPs are underlined and the predicted subcellular locations of GmCYPs are shown by colors as indicated.

To explore the evolutionary relationship among soybean CYPs, a phylogenetic analysis was performed using their predicted amino acid sequences (Figure 3). As observed for many other genes in soybean, most of the predicted GmCYPs clustered together in pairs, reflecting the ancient genome duplication event [27,35]. Such events result in two copies of each gene which undergo shuffling and rearrangement, creating the potential for new diversity. There are four possible fates of duplicated genes [36]. First, one copy of the gene may be deleted during the course of evolution, resulting in loss of functional redundancy. Second, both copies of the genes may be retained and share the ancestral function, but gradually develop partially different functions (sub-functionalization). Third, one copy of the gene may acquire new function(s) during the course of evolution (neo-functionalization). Finally, there may be an intermediate stage between sub- and neo-functionalization. Which of these outcomes occur depends on the role of the specific gene in plant growth and development. Only those genes that are associated with critical functions for normal plant growth and development are retained, while others may be lost. The large number of *CYP*s present in the soybean genome thus likely reflects a combination of duplication and the important role of *GmCYPs* in soybean during normal growth and development, as well as in response to environmental stimuli.

Of the 62 GmCYPs, 54 are clustered in pairs (27 pairs) in the phylogenetic tree. The remaining 8 GmCYPs branched-off from the terminal branch of another pair of GmCYPs. This analysis further revealed that the multi-domain GmCYPs cluster together.

We also attempted to correlate the clustering of GmCYPs in the phylogenetic tree with their predicted subcellular localization. Interestingly, the GmCYPs predicted to be targeted to the same subcellular compartment grouped together as a separate clade. For example, GmCYPs with chloroplast transit peptide (GmCYP10, GmCYP23, GmCYP14, GmCYP28 and GmCYP12) formed a distinct clade on the tree. Another 4 chloroplast-localizing GmCYPs (GmCYP48, GmCYP52, GmCYP53, and GmCYP60) also formed a distinct clade, but in a different location on the tree. Similarly, the GmCYPs with nuclear localization signal (GmCYP27, GmCYP36, and GmCYP54, GmCYP43, GmCYP46) also formed separate clades on the tree (Figure 3). A similar pattern of gene clustering was observed for the GmCYPs predicted to localize in mitochondria or that were secretory.

By comparing the positions of *GmCYPs* on the chromosome map (Figure 2) and in the phylogenetic tree (Figure 3), an interesting grouping pattern was observed. If the *GmCYPs* were localized together on a chromosome, their paralogs were also found together on a different chromosome. For example, *GmCYP4, GmCYP39,* and *GmCYP46* are clustered at the sub-telomere region of chromosome 4, and are most similar to *GmCYP3, GmCYP38,* and *GmCYP43*, respectively, which are clustered together in the sub-telomere region of chromosome 6 (Figure 2). Similarly, *GmCYP36, GmCYP13,* and *GmCYP7* (chromosome 3) paired with *GmCYP27, GmCYP15,* and *GmCYP11,* respectively, from chromosome 19, and *GmCYP18* and *GmCYP32* (chromosome 11) paired with *GmCYP19* and *GmCYP31*, respectively (chromosome 1), whereas *GmCYP1* and *GmCYP41* (chromosome 11) paired with *GmCYP2* and *GmCYP40* (chromosome 12). Moreover, *GmCYP34* and *GmCYP26*, from chromosome 11, paired up with *GmCYP24* and *GmCYP25*, respectively, from chromosome 18. These findings provide strong evidence for segmental duplication of chromosomal regions containing the *GmCYPs*, such as has been shown to play a vital role in the evolutionary generation of members of other gene families [37,38].

### Gene structures of *GmCYPs*

**Figure 4:**
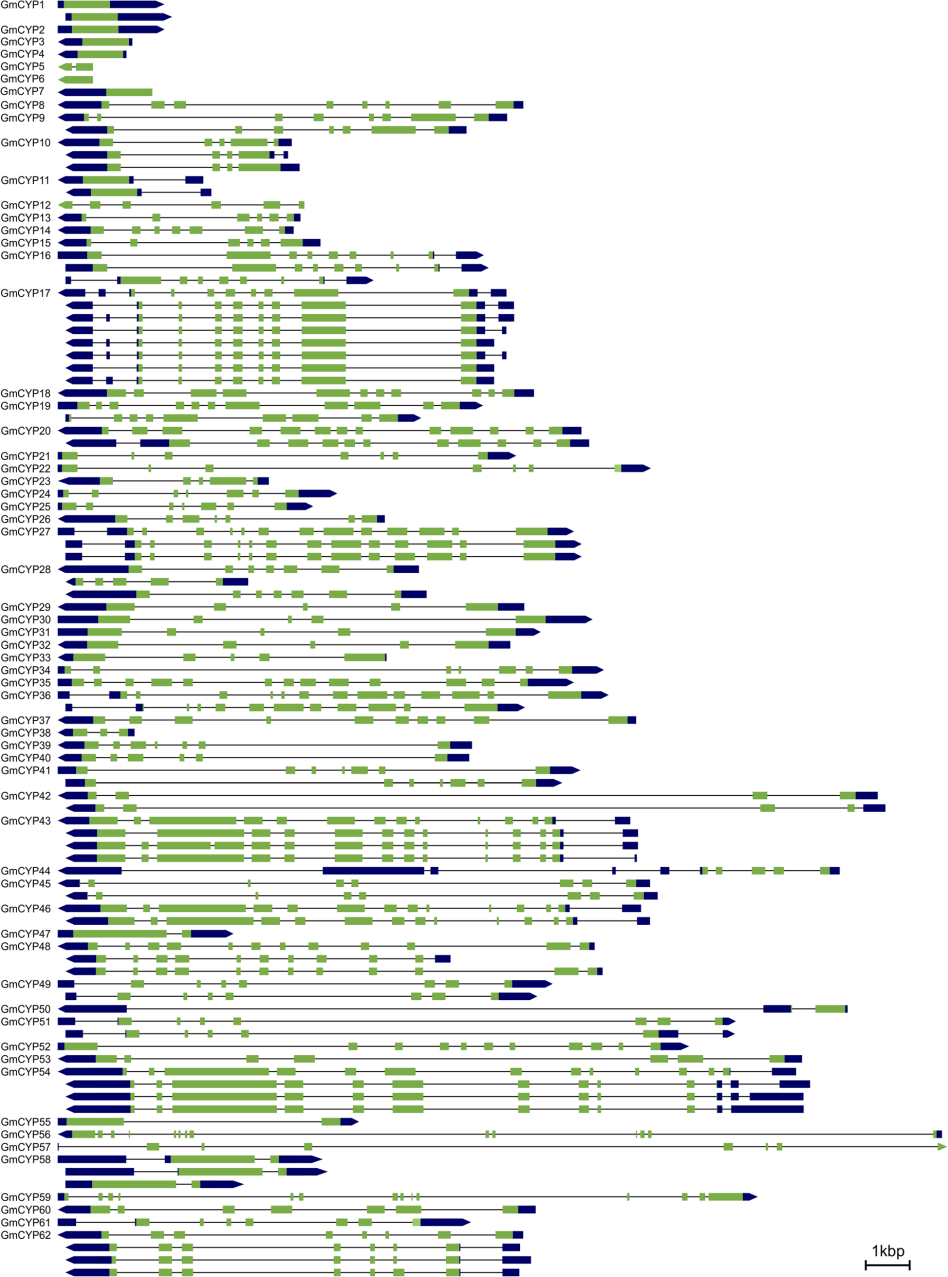
**Schematic diagrams of the exon-intron structures, and splice variants of** ***GmCYPs*** **.** Exon-intron structures of *GmCYPs* were compiled from Phyotozome database (http://phytozome.jgi.doe.gov/pz/portal.html#!info?alias=Org_Gmax). *GmCYP* with predicted alternate transcripts are shown below the corresponding genes. The green boxes, black boxes and lines indicate exons, UTRs and introns, respectively. Left to right direction of transcript shows “+” strand while right to left shows “-” strand, relative to the annotation of the genome sequence. Gene structure images are drawn to scale except for *GmCYP50*, *GmCYP56*, and *GmCYP59*, where diagrams are reduced to 0.5X, 0.35X, and 0.5X, respectively.

Analysis of the exon-intron structure of the *GmCYP* genes showed several variations (Figure 4). Six *GmCYP* genes (*GmCYP1- GmCYP4*, *GmCYP6* and *GmCYP7*) contained no intron in their open reading frame (ORF). The number of introns varied from 1 to 13 in the ORFs of other *GmCYP* genes. The *GmCYP5, GmCYP47, GmCYP50, GmCYP55* and *GmCYP58* contained a single intron in their ORF while the largest numbers of introns were found in *GmCYP56*. The size of intron also varied considerably between different *GmCYP* gene family members with their size ranging from 39 bp (*GmCYP5*) to 9359 bp (*GmCYP56*) in the primary transcripts. Several other genes such as *GmCYP22, GmCYP30, GmCYP34, GmCYP39-GmCYP42, GmCYP45, GmCYP49, GmCYP51- GmCYP53, GmCYP55- GmCYP57* and *GmCYP59* contained introns larger than 4.0 kb in their ORFs. It has been suggested that the genome size may be correlated with intron size and that some elements of genome size evolution occurs within the gene [39]. However, in *Gossypium sps.,* intron and genome size evolution are not coupled [40]. In the regions of low recombination, longer introns are selectively advantageous as they improve recombination and possibly counterbalance the mutational bias towards deletion [41]. A large-scale comparative analysis of intron positions among different kingdoms (animal, plant and fungus) identified a large number of positions that are likely to be ancestral [42]. Analysis of intron sizes and positions in paralogs among *GmCYP* family did not show any specific pattern. However, in the majority of cases, the exon-intron numbers were similar in the genes that clustered together in the phylogenetic tree (Figure 3), for example, *GmCYP25* and *GmCYP26* or *GmCYP47* and *GmCYP58* or *GmCYP20* and *GmCYP35*. The 5′ and 3′ untranslated regions (UTR) that border protein-coding sequences are important structural and regulatory elements of eukaryotic genes [43] and also contain large numbers of introns [44]. Out of 62 *GmCYPs*, 12 contained a single intron in the 5′UTR region while remaining *GmCYPs* consisted of intronless 5′UTR. The 3′UTR of two *GmCYPs, GmCYP16* and *GmCYP50,* were interrupted by a single intron whereas *GmCYP17* and *GmCYP44* contained 2 and 5 introns, respectively. The number of exon and intron in each *GmCYP* gene is shown in Additional file 2.

### Expression analysis of *GmCYP* genes

**Figure 5:**
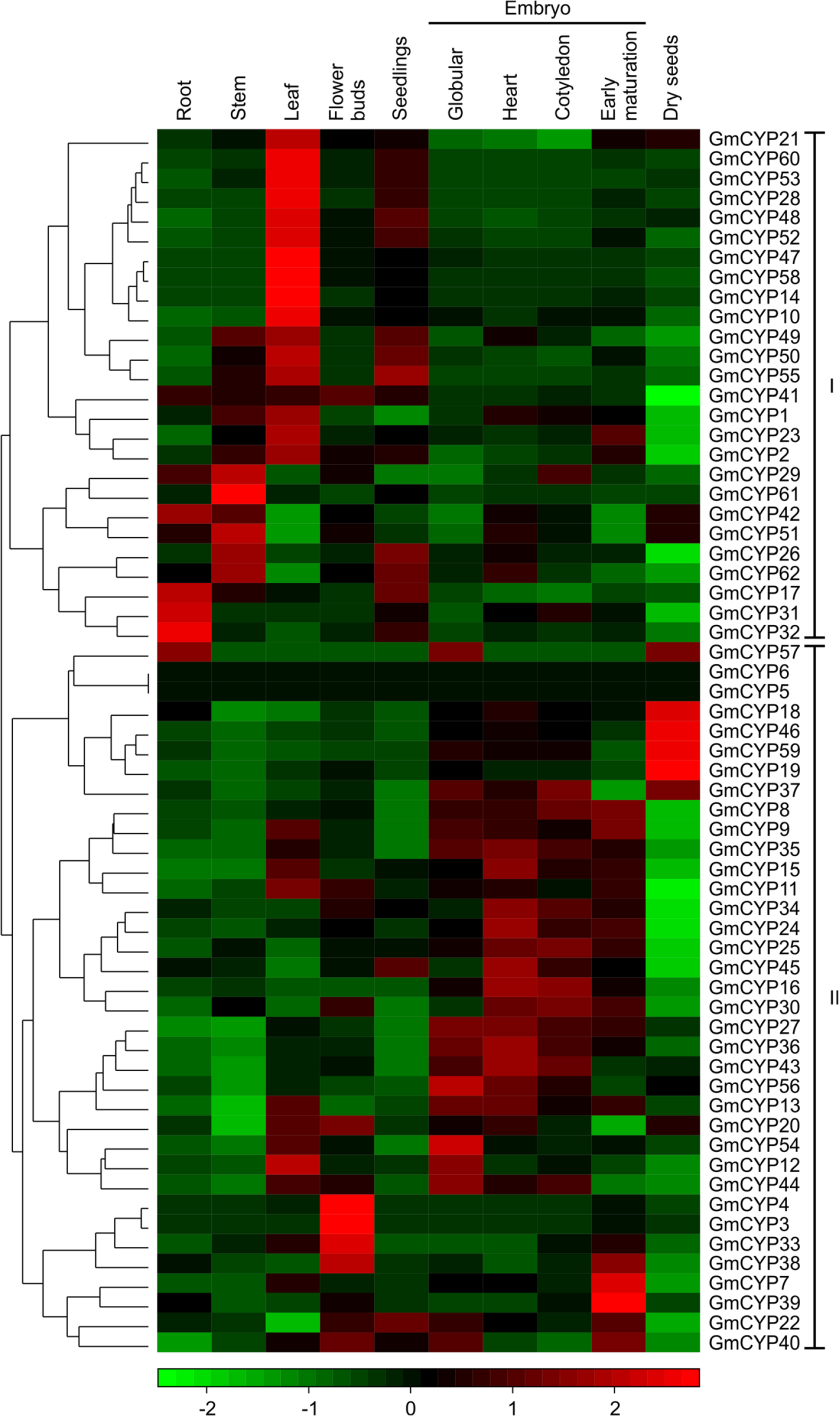
**Expression analyses of soybean** ***CYP*** **genes.** The transcriptome data of soybean across different tissues and developmental stages were obtained from the National Center for Biotechnology Information (http://www.ncbi.nlm.nih.gov/geo/query/acc.cgi?acc=GSE29163) for heatmap generation. The color scale below the heat map indicates expression values, green indicating low transcript abundance and red indicating high levels of transcript abundance. Clustering of *GmCYPs* in **(I)** vegetative tissue or **(II)** reproductive or seed tissue is shown.

To determine expression patterns of *GmCYP* genes, we used publicly-available genome-wide transcript profiling data of soybean tissues as a resource (http://www.ncbi.nlm.nih.gov/geo/query/acc.cgi?acc=GSE29163). The dataset contains RNAseq reads from soybean seeds across several stages of seed development (globular, heart, cotyledon, early-maturation, dry), and reproductive (floral buds) and vegetative (leaves, roots, stems, seedlings) tissues. As shown in Figure 5, most of the *GmCYP* genes showed distinct tissue-specific expression pattern. Out of the 62 *GmCYP* genes, 26 were expressed in the vegetative tissues whereas 34 were expressed in floral buds and different stages of seed development. Two *GmCYPs*, *GmCYP5* and *GmCYP6,* contained no sequence read in any of the soybean tissues included in the study. In addition, there were no EST or TC sequences in the DFCI gene index database with a perfect match to *GmCYP5* and *GmCYP6* (Additional file 1). These evidences indicated that *GmCYP5* and *GmCYP6* are pseudogenes or expressed under special conditions or at specific developmental stages. The gene expression data revealed that the majority of *GmCYPs* (41%) were expressed in leaf tissue with the highest transcript accumulation level. Furthermore, it is interesting to note that the GmCYPs predicted to localize in the chloroplast were expressed in leaf tissues, suggesting their possible role in photosynthesis. Expression of several *GmCYP* genes in seed tissues during development indicates an important role of these genes in seed development.

## Conclusions

Taken together, we have performed a comprehensive sequence analysis of soybean *CYP* genes (*GmCYPs*), and provided detailed information on them. Specifically, our results show that the soybean genome contains 62 *CYP* genes, the largest *CYP* gene family identified in any organism to date. The presence of predicted motifs, subcellular localization and their sequence homology with other identified CYPs from other organisms provided insight into their putative function. Results of the present study indicate a genome-wide segmental and tandem duplication during expansion of the *GmCYP* gene family.

## Methods

### Database search for *CYP* genes in soybean

To identify all the *CYPs* present in the soybean genome, the nucleotide sequence of the *GmCYP1* (GenBank:AF456323) was used for a BLASTN [45] query against the new soybean genome database (Wm82.a2.v1) (http://phytozome.jgi.doe.gov/pz/portal.html) [46]. The newly identified sequences were subsequently used as queries to find other less similar *GmCYP*s. The chromosomal locations for all *GmCYPs* were obtained from the soybean genome database to draw the chromosomal map. The molecular weight for each GmCYP was calculated using ProtParam software [47] (http://web.expasy.org/protparam/). TargetP1 [48] (http://www.cbs.dtu.dk/services/TargetP/) and WoLF-PSORT [49] (http://wolfpsort.org/) were used to identify putative sub-cellular localization of the predicted protein sequences, and domain information was obtained from the soybean genome database [27]. To identify the transcribed *GmCYP*s in soybean, the coding sequence of each *GmCYP* was used as a query to BLAST against the soybean gene index (http://compbio.dfci.harvard.edu/tgi/). The Tentative Contig (TC) sequences in the soybean gene index database were aligned with the corresponding *GmCYP* sequences to identify the percentage identity and coverage. Similarly, to find the GmCYP orthologs in *Arabidopsis*, the amino acid sequences of GmCYPs were used as queries to BLAST against the *Arabidopsis* protein database (http://www.arabidopsis.org/) [50].

### Multiple sequence alignment and phylogenetic analyses

To investigate the phylogenetic relationships among GmCYP proteins, and their molecular evolution, a phylogenetic tree was generated. Multiple sequence alignment of the deduced amino acid sequences of all GmCYP proteins were aligned by Clustal X and the alignment was imported into MEGA5.1 to create a phylogenetic tree [51]. Neighbour-Joining method was used with 1000 bootstrap replicates. The tree was exported into the Interactive Tree Of Life (http://itol.embl.de) for annotation and manipulation [52].

### Expression analysis of soybean *CYP* genes

To determine the expression patterns of *CYP* genes in soybean tissues, the publically available transcriptome data (http://www.ncbi.nlm.nih.gov/geo/query/acc.cgi?acc=GSE29163) was used as a main source. The illumina sequencing of transcripts from ten different soybean tissues were downloaded from the NCBI database (http://www.ncbi.nlm.nih.gov/) with accession numbers SRX062325-SRX062334. After normalization of the dataset, the value of each gene was centered by subtracting the mean normalized value for each gene and scaled by dividing the centered value by the standard deviation of the gene following Eisen et al. [53]. The heatmap for *GmCYP* genes was generated in R using the heatmap.2 function from the gplots CRAN library (http://CRAN.R-project.org/package=gplots).

## Availability of supporting data

Phylogenetic data (tree and data used to generate them) have been deposited in TreeBASE repository and is available under the URL http://purl.org/phylo/treebase/phylows/study/TB2:S16455.

## Additional files

Additional file 1:
**Soybean** ***cyclophilin*** **gene family.**
Additional file 2:
**Number of exon/introns and splice variants in** ***GmCYP*** **genes.**
