# Supplementary material for: Genome-wide analysis of Cyclophilin gene family in soybean (Glycine max)
Source: BMC Plant Biol. 2014 Oct 29;14:282. doi: 10.1186/s12870-014-0282-7 (PMC4220052; doi:10.1186/s12870-014-0282-7)
Supplement: Additional file 1: — Soybean cyclophilin gene family. [file 12870_2014_282_MOESM1_ESM.docx]

| **Table S1: Soybean Cyclophilin Gene Family** | | | | | | | | | | |
| --- | --- | --- | --- | --- | --- | --- | --- | --- | --- | --- |
| **Gene Name** | **Locus Name** | **Gene Location** | **Splice variant(s)** | **Transcript size (bp)** | **Coding sequence (bp)** | **Protein Molecular weight (kDa)** | **Sub cellular Location** | **Domain information** | **Corresponding TC number (% Identity)** | **Best *Arabidopsis* TAIR10 hit** |
| GmCYP1 | Glyma.11G098700 | Chr11: 7504475 - 7505686 | 1 | 973 | 519 | 18.22 | Cytosol | SD | TC464530 (100) | [AT2G16600.1](http://arabidopsis.org/servlets/TairObject?type=gene&name=AT2G16600.1) |
| GmCYP2 | Glyma.12G024700 | Chr12: 1807036 - 1808259 | 1 | 1224 | 519 | 18.12 | Cytosol | SD | TC422035 (100) | [AT2G16600.1](http://arabidopsis.org/servlets/TairObject?type=gene&name=AT2G16600.1) |
| GmCYP3 | Glyma.06G005100 | Chr06: 431771 - 432624 | 1 | 854 | 519 | 18.193 | Cytosol | SD | TC428777 (100) | [AT2G16600.1](http://arabidopsis.org/servlets/TairObject?type=gene&name=AT2G16600.1) |
| GmCYP4 | Glyma.04G005300 | Chr04: 439283 - 440057 | 1 | 775 | 519 | 18.197 | Cytosol | SD | TC428777 (94) | [AT2G16600.1](http://arabidopsis.org/servlets/TairObject?type=gene&name=AT2G16600.1) |
| GmCYP5 | Glyma.02G021800 | Chr02: 1930733 - 1931125 | 1 | 354 | 354 | 13.234 | Cytosol | SD | TC464530 (84) | [AT2G16600.1](http://arabidopsis.org/servlets/TairObject?type=gene&name=AT2G16600.1) |
| GmCYP6 | Glyma.15G242500 | Chr15: 46274671 - 46275063 | 1 | 393 | 393 | 14.808 | Cytosol | SD | TC422035 (80) | [AT2G16600.1](http://arabidopsis.org/servlets/TairObject?type=gene&name=AT2G16600.1) |
| GmCYP7 | Glyma.03G251600 | Chr03: 44735415 - 44736486 | 1 | 1072 | 528 | 18.884 | Cytosol | SD | TC429055 (100) | [AT3G56070.1](http://arabidopsis.org/servlets/TairObject?type=gene&name=AT3G56070.1) |
| GmCYP8 | Glyma.05G080800 | Chr05: 11891126 - 11894777 | 1 | 1611 | 1083 | 40.015 | Cytosol | MD | TC443482 (100) | [AT2G15790.1](http://arabidopsis.org/servlets/TairObject?type=gene&name=AT2G15790.1) |
| GmCYP9 | Glyma.17G177600 | Chr17: 19104479 - 19109551 | 2 | 1241 | 1083 | 40.12 | Cytosol | MD | TC443482 (95)* | [AT2G15790.1](http://arabidopsis.org/servlets/TairObject?type=gene&name=AT2G15790.1) |
| GmCYP10 | Glyma.20G249300 | Chr20: 47818406 - 47821050 | 3 | 1380 | 762 | 27.103 | Chloroplast | SD | TC462161 (100) | [AT5G13120.1](http://arabidopsis.org/servlets/TairObject?type=gene&name=AT5G13120.1) |
| GmCYP11 | Glyma.19G249000 | Chr19: 49513162 - 49514810 | 2 | 1062 | 528 | 18.955 | Cytosol | SD | TC422384 (100) | [AT3G56070.1](http://arabidopsis.org/servlets/TairObject?type=gene&name=AT3G56070.1) |
| GmCYP12 | Glyma.03G225600 | Chr03: 42790892 - 42793688 | 1 | 711 | 711 | 25.973 | Chloroplast | SD | TC428054 (94) | [AT3G62030.3](http://arabidopsis.org/servlets/TairObject?type=gene&id=1000688525) |
| GmCYP13 | Glyma.03G198200 | Chr03: 40759912 - 40762652 | 1 | 793 | 495 | 18.153 | Cytosol | SD | TC425919 (100) | [AT2G36130.1](http://arabidopsis.org/servlets/TairObject?type=gene&name=AT2G36130.1) |
| GmCYP14 | Glyma.19G222600 | Chr19: 47480824 - 47483494 | 1 | 1253 | 783 | 28.32 | Chloroplast | SD | TC428054 (100) | [AT3G62030.3](http://arabidopsis.org/servlets/TairObject?type=gene&name=AT3G62030.3) |
| GmCYP15 | Glyma.19G196100 | Chr19: 45334576 - 45337557 | 1 | 1200 | 666 | 24.924 | Cytosol | SD | TC430194 (99)* | [AT2G36130.1](http://arabidopsis.org/servlets/TairObject?type=gene&id=34868) |
| GmCYP16 | Glyma.07G210200 | Chr07: 38085486 - 38090339 | 3 | 1745 | 1086 | 40.247 | Cytosol | MD | TC460136 (97)* | [AT2G15790.1](http://arabidopsis.org/servlets/TairObject?type=gene&name=AT2G15790.1) |
| GmCYP17 | Glyma.02G134800 | Chr02: 13958441 - 13963538 | 8 | 1770 | 1086 | 40.205 | Cytosol | MD | TC460136 (100) | [AT2G15790.1](http://arabidopsis.org/servlets/TairObject?type=gene&name=AT2G15790.1) |
| GmCYP18 | Glyma.11G047800 | Chr11: 3568992 - 3574381 | 1 | 2576 | 1794 | 65.371 | Nucleus | MD | TC421742 (96)* | [AT5G67530.1](http://arabidopsis.org/servlets/TairObject?type=gene&name=AT5G67530.1) |
| GmCYP19 | Glyma.01G194100 | Chr01: 52844224 - 52849031 | 2 | 2292 | 1794 | 65.235 | Nucleus | MD | TC421742 (97)* | [AT5G67530.1](http://arabidopsis.org/servlets/TairObject?type=gene&name=AT5G67530.1) |
| GmCYP20 | Glyma.13G318300 | Chr13: 41269055 - 41274970 | 2 | 2554 | 1851 | 69.361 | Nucleus | MD | TC458065 (99)* | [AT3G44600.1](http://arabidopsis.org/servlets/TairObject?type=gene&name=AT3G44600.1) |
| GmCYP21 | Glyma.09G090900 | Chr09: 12216949 - 12222137 | 1 | 967 | 194 | 20.965 | Cytosol | SD | TC422604 (99)* | [AT2G38730.1](http://arabidopsis.org/servlets/TairObject?type=gene&name=AT2G38730.1) |
| GmCYP22 | Glyma.13G193500 | Chr13: 30669375 - 30676126 | 1 | 947 | 183 | 19.748 | Cytosol | SD | TC422604 (99) | [AT2G38730.1](http://arabidopsis.org/servlets/TairObject?type=gene&id=36586) |
| GmCYP23 | Glyma.10G298200 | Chr10: 51484362 - 51486738 | 1 | 1349 | 251 | 26.904 | Chloroplast | SD | TC465254 (100) | [AT5G13120.1](http://arabidopsis.org/servlets/TairObject?type=gene&id=136773) |
| GmCYP24 | Glyma.18G062900 | Chr18: 5757258 - 5760418 | 1 | 1118 | 204 | 21.954 | Secretory | SD | TC451996 (100) | [AT5G58710.1](http://arabidopsis.org/servlets/TairObject?type=gene&id=136647) |
| GmCYP25 | Glyma.18G027400 | Chr18: 2060386 - 2063284 | 1 | 1061 | 235 | 25.72 | Secretory | SD | TC459271 (99)* | [AT3G55920.1](http://arabidopsis.org/servlets/TairObject?type=gene&name=AT3G55920.1) |
| GmCYP26 | Glyma.11G229800 | Chr11: 32512707 - 32516411 | 1 | 1459 | 238 | 25.831 | Secretory | SD | TC470206 (100) | [AT3G55920.1](http://arabidopsis.org/servlets/TairObject?type=gene&name=AT3G55920.1) |
| GmCYP27 | Glyma.19G160200 | Chr19: 42107601 - 42113486 | 3 | 2693 | 659 | 73.522 | Nucleus | SD | TC461722 (99)* | [AT3G63400.1](http://arabidopsis.org/servlets/TairObject?type=gene&name=AT3G63400.1) |
| GmCYP28 | Glyma.10G139500 | Chr10: 37323333 - 37327399 | 3 | 1869 | 263 | 29.017 | Chloroplast | SD | TC424043 (99)* | [AT3G62030.3](http://arabidopsis.org/servlets/TairObject?type=gene&name=AT3G62030.3) |
| GmCYP29 | Glyma.18G248100 | Chr18: 53511479 - 53516777 | 1 | 1822 | 326 | 36.427 | secretory | SD | TC459175 (96)* | [AT4G17070.1](http://arabidopsis.org/servlets/TairObject?type=gene&id=130215) |
| GmCYP30 | Glyma.09G245200 | Chr09: 46732987 - 46739075 | 1 | 1988 | 327 | 36.694 | Secretory | SD | TC459175 (96)* | [AT4G17070.1](http://arabidopsis.org/servlets/TairObject?type=gene&name=AT4G17070.1) |
| GmCYP31 | Glyma.01G204300 | Chr01: 53712020 - 53717501 | 1 | 1645 | 337 | 37.089 | PM/Mitochondria# | SD | TC428737(96)* | [AT4G17070.1](http://arabidopsis.org/servlets/TairObject?type=gene&name=AT4G17070.1) |
| GmCYP32 | Glyma.11G038700 | Chr11: 2761557 - 2766671 | 1 | 1595 | 337 | 37.009 | Secretory | SD | TC428737 (99)* | [AT4G17070.1](http://arabidopsis.org/servlets/TairObject?type=gene&name=AT4G17070.1) |
| GmCYP33 | Glyma.17G148700 | Chr17: 12315782 - 12319509 | 1 | 1301 | 373 | 41.633 | Secretory | SD | TC433463 (85) | [AT4G17070.1](http://arabidopsis.org/servlets/TairObject?type=gene&id=130215) |
| GmCYP34 | Glyma.11G175200 | Chr11: 19229203 - 19235404 | 1 | 1065 | 204 | 21.944 | Secretory | SD | TC423512 (100) | [AT2G29960.1](http://arabidopsis.org/servlets/TairObject?type=gene&name=AT2G29960.1) |
| GmCYP35 | Glyma.12G182700 | Chr12: 34377747 - 34383622 | 1 | 2543 | 616 | 69.326 | Cytosol | MD | TC458065 (99)* | [AT3G44600.1](http://arabidopsis.org/servlets/TairObject?type=gene&name=AT3G44600.1) |
| GmCYP36 | Glyma.03G157900 | Chr03: 37332635 - 37338878 | 2 | 2559 | 668 | 74.261 | Nucleus | SD | TC461219 (97)* | [AT3G63400.1](http://arabidopsis.org/servlets/TairObject?type=gene&name=AT3G63400.1) |
| GmCYP37 | Glyma.17G218800 | Chr17: 37027171 - 37033720 | 2 | 1982 | 493 | 55.642 | Cytosol | SD | TC443202 (100)* | [AT4G33060.1](http://arabidopsis.org/servlets/TairObject?type=gene&name=AT4G33060.1) |
| GmCYP38 | Glyma.06G004000 | Chr06: 368126 - 368994 | 1 | 582 | 114 | 12.359 | Cytosol | SD | TC446906 (86) | [AT4G34960.1](http://arabidopsis.org/servlets/TairObject?type=gene&id=432408) |
| GmCYP39 | Glyma.04G004300 | Chr04: 373953 - 378634 | 2 | 1233 | 232 | 25.520 | Secretory | SD | EV278562 (100)* | [AT4G34960.1](http://arabidopsis.org/servlets/TairObject?type=gene&name=AT4G34960.1) |
| GmCYP40 | Glyma.12G031500 | Chr12: 2354359 - 2360272 | 1 | 1264 | 236 | 26.182 | Secretory | SD | TC446906 (100)* | [AT4G34960.1](http://arabidopsis.org/servlets/TairObject?type=gene&name=AT4G34960.1) |
| GmCYP41 | Glyma.11G106400 | Chr11: 8108697 - 8114349 | 1 | 1238 | 236 | 26.151 | Secretory | SD | TC428887 (100) | [AT4G34960.1](http://arabidopsis.org/servlets/TairObject?type=gene&name=AT4G34960.1) |
| GmCYP42 | Glyma.01G144800 | Chr01: 47935477 - 47944771 | 2 | 1087 | 165 | 18.082 | Cytosol | SD | TC429931 (100)* | [AT1G01940.1](http://arabidopsis.org/servlets/TairObject?type=gene&name=AT1G01940.1) |
| GmCYP43 | Glyma.06G070300 | Chr06: 5381397 - 5387924 | 4 | 3138 | 850 | 95.935 | Nucleus | SD | TC450817 (94) | [AT4G32420.1](http://arabidopsis.org/servlets/TairObject?type=gene&id=129751) |
| GmCYP44 | Glyma.01G044900 | Chr01: 5023297 - 5032196 | 1 | 2766 | 167 | 18.707 | Secretory | SD | TC438717 (99) | [AT1G26940.1](http://arabidopsis.org/servlets/TairObject?type=gene&id=138748) |
| GmCYP45 | Glyma.02G105500 | Chr02: 10019490 - 10026235 | 2 | 1085 | 226 | 25.613 | Secretory | SD | TC479526 (100) | [AT1G26940.1](http://arabidopsis.org/servlets/TairObject?type=gene&name=AT1G26940.1) |
| GmCYP46 | Glyma.04G068700 | Chr04: 5734490 - 5741111 | 2 | 2532 | 843 | 94.964 | Nucleus | SD | TC450817 (89) | [AT4G32420.1](http://arabidopsis.org/servlets/TairObject?type=gene&id=129751) |
| GmCYP47 | Glyma.05G014300 | Chr05: 1339886 - 1341894 | 1 | 1836 | 387 | 42.0186 | Chloroplast | SD | TC435070 (99)* | [AT1G74070.1](http://arabidopsis.org/servlets/TairObject?type=gene&name=AT1G74070.1) |
| GmCYP48 | Glyma.06G249300 | Chr06: 42115744 - 42121879 | 3 | 1751 | 439 | 47.320 | Chloroplast | SD | TC429384 (97)* | [AT3G15520.1](http://arabidopsis.org/servlets/TairObject?type=gene&name=AT3G15520.1) |
| GmCYP49 | Glyma.07G157800 | Chr07: 19488288 - 19493877 | 2 | 1324 | 225 | 25.689 | Mitochondria | SD | TC482811 (80) | [AT3G66654.1](http://arabidopsis.org/servlets/TairObject?type=gene&id=42019) |
| GmCYP50 | Glyma.09G095400 | Chr09: 14232104 - 14249951 | 1 | 2922 | 227 | 24.599 | Chloroplast | SD | TC493586 (95)* | [AT5G35100.1](http://arabidopsis.org/servlets/TairObject?type=gene&id=132788) |
| GmCYP51 | Glyma.10G206100 | Chr10: 43763030 - 43770594 | 2 | 947 | 232 | 26.174 | Mitochondria | SD | TC482811 (99)* | [AT3G66654.1](http://arabidopsis.org/servlets/TairObject?type=gene&name=AT3G66654.1) |
| GmCYP52 | Glyma.12G148600 | Chr12: 20934278 - 20941440 | 1 | 1724 | 439 | 47.508 | Chloroplast | SD | TC429384 (94) | [AT3G15520.1](http://arabidopsis.org/servlets/TairObject?type=gene&name=AT3G15520.1) |
| GmCYP53 | Glyma.13G169500 | Chr13: 28348181 - 28356641 | 1 | 1983 | 445 | 48.730 | Chloroplast | SD | TC431861 (96)* | [AT3G01480.1](http://arabidopsis.org/servlets/TairObject?type=gene&name=AT3G01480.1) |
| GmCYP54 | Glyma.14G125200 | Chr14: 19828597 - 19836960 | 4 | 3559 | 849 | 96.167 | Nucleus | SD | TC471889 (100)* | [AT4G32420.1](http://arabidopsis.org/servlets/TairObject?type=gene&id=129751) |
| GmCYP55 | Glyma.15G202300 | Chr15: 25414464 - 25417891 | 1 | 1175 | 286 | 31.234 | Chloroplast | SD | TC461630 (99)* | [AT5G35100.1](http://arabidopsis.org/servlets/TairObject?type=gene&name=AT5G35100.1) |
| GmCYP56 | Glyma.15G213200 | Chr15: 33587471 - 33615806 | 1 | 2537 | 633 | 74.013 | Nucleus | MD | TC481910 (98)* | [AT1G53720.1](http://arabidopsis.org/servlets/TairObject?type=gene&name=AT1G53720.1) |
| GmCYP57 | Glyma.15G231800 | Chr15: 43588806 - 43598902 | 1 | 546 | 181 | 19.905 | Cytosol | SD | TC422604 (95) | [AT2G38730.1](http://arabidopsis.org/servlets/TairObject?type=gene&id=36586) |
| GmCYP58 | Glyma.17G122500 | Chr17: 9801722 - 9804711 | 3 | 2374 | 350 | 38.183 | Chloroplast | SD | TC435070 (94) | [AT1G74070.1](http://arabidopsis.org/servlets/TairObject?type=gene&name=AT1G74070.1) |
| GmCYP59 | Glyma.19G004200 | Chr19: 338885 - 354769 | 1 | 2403 | 640 | 74.699 | Nucleus | MD | TC485676 (99)* | [AT1G53720.1](http://arabidopsis.org/servlets/TairObject?type=gene&id=30353) |
| GmCYP60 | Glyma.19G009700 | Chr19: 907630 - 913061 | 1 | 1921 | 445 | 48.731 | Chloroplast | SD | TC431861 (99)* | [AT3G01480.1](http://arabidopsis.org/servlets/TairObject?type=gene&name=AT3G01480.1) |
| GmCYP61 | Glyma.20G005600 | Chr20: 532311 - 536994 | 1 | 1493 | 230 | 25.696 | Mitochondria | SD | TC452908 (85) | [AT3G66654.1](http://arabidopsis.org/servlets/TairObject?type=gene&id=42019) |
| GmCYP62 | Glyma.20G184400 | Chr20: 42259262 - 42264549 | 4 | 1489 | 292 | 33.172 | Mitochondria | SD | TC452908 (99)* | [AT3G66654.1](http://arabidopsis.org/servlets/TairObject?type=gene&name=AT3G66654.1) |

SD, single domain; MD, multi-domain; #, prediction with less confidence; *, contigs with >95% sequence identity but with query coverage <100%; Underline, contigs with 99-100% sequence identity with 100% sequence coverage.

- Subcellular localization prediction software’s references: Target P (PMID: 17446895), PSORT(PMID: 10087920) and WOLF-PSORT (PMID:17517783),
