# Supplementary material for: Genome-wide analysis of Cyclophilin gene family in soybean (Glycine max)
Source: BMC Plant Biol. 2014 Oct 29;14:282. doi: 10.1186/s12870-014-0282-7 (PMC4220052; doi:10.1186/s12870-014-0282-7)
Supplement: Additional file 2: — Number of exon/introns and splice variants in GmCYP genes. [file 12870_2014_282_MOESM2_ESM.docx]

| **Table S2 Number of exon/introns and splice variants in *GmCYP* genes.** | | | | | |
| --- | --- | --- | --- | --- | --- |
| Gene | No. of transcripts | No. of exons | No. of introns | | |
|  |  |  | CDS | 5'UTR | 3'UTR |
| *GmCYP1* | 1 | 1 | 0 | 0 | 0 |
| *GmCYP2* | 1 | 1 | 0 | 0 | 0 |
| *GmCYP3* | 1 | 1 | 0 | 0 | 0 |
| *GmCYP4* | 1 | 1 | 0 | 0 | 0 |
| *GmCYP5* | 1 | 2 | 1 | 0 | 0 |
| *GmCYP6* | 1 | 1 | 0 | 0 | 0 |
| *GmCYP7* | 1 | 1 | 0 | 0 | 0 |
| *GmCYP8* | 1 | 8 | 7 | 0 | 0 |
| *GmCYP9* | 2 | 8 | 7 | 0 | 0 |
| *GmCYP10* | 3 | 5 | 4 | 0 | 0 |
| *GmCYP11* | 2 | 1 | 0 | 1 | 0 |
| *GmCYP12* | 1 | 6 | 5 | 0 | 0 |
| *GmCYP13* | 1 | 6 | 5 | 0 | 0 |
| *GmCYP14* | 1 | 7 | 6 | 0 | 0 |
| *GmCYP15* | 1 | 6 | 5 | 0 | 0 |
| *GmCYP16* | 3 | 8 | 7 | 0 | 1 |
| *GmCYP17* | 8 | 8 | 7 | 1 | 2 |
| *GmCYP18* | 1 | 11 | 10 | 0 | 0 |
| *GmCYP19* | 2 | 11 | 10 | 0 | 0 |
| *GmCYP20* | 2 | 13 | 12 | 0 | 0 |
| *GmCYP21* | 1 | 7 | 6 | 0 | 0 |
| *GmCYP22* | 1 | 7 | 6 | 0 | 0 |
| *GmCYP23* | 1 | 5 | 4 | 0 | 0 |
| *GmCYP24* | 1 | 7 | 6 | 0 | 0 |
| *GmCYP25* | 1 | 7 | 6 | 0 | 0 |
| *GmCYP26* | 1 | 7 | 6 | 0 | 0 |
| *GmCYP27* | 3 | 13 | 12 | 1 | 0 |
| *GmCYP28* | 3 | 7 | 6 | 0 | 0 |
| *GmCYP29* | 1 | 5 | 4 | 0 | 0 |
| *GmCYP30* | 1 | 5 | 4 | 0 | 0 |
| *GmCYP31* | 1 | 5 | 4 | 0 | 0 |
| *GmCYP32* | 1 | 5 | 4 | 0 | 0 |
| *GmCYP33* | 1 | 5 | 4 | 0 | 0 |
| *GmCYP34* | 1 | 7 | 6 | 0 | 0 |
| *GmCYP35* | 1 | 13 | 12 | 0 | 0 |
| *GmCYP36* | 2 | 13 | 12 | 1 | 0 |
| *GmCYP37* | 2 | 10 | 9 | 0 | 0 |
| *GmCYP38* | 1 | 3 | 2 | 0 | 0 |
| *GmCYP39* | 2 | 7 | 6 | 0 | 0 |
| *GmCYP40* | 1 | 7 | 6 | 0 | 0 |
| *GmCYP41* | 1 | 7 | 6 | 0 | 0 |
| *GmCYP42* | 2 | 4 | 3 | 0 | 0 |
| *GmCYP43* | 4 | 13 | 12 | 1 | 0 |
| *GmCYP44* | 1 | 5 | 4 | 0 | 5 |
| *GmCYP45* | 2 | 8 | 7 | 0 | 0 |
| *GmCYP46* | 2 | 13 | 12 | 1 | 0 |
| *GmCYP47* | 1 | 2 | 1 | 0 | 0 |
| *GmCYP48* | 3 | 12 | 11 | 0 | 0 |
| *GmCYP49* | 2 | 7 | 6 | 1 | 0 |
| *GmCYP50* | 1 | 2 | 1 | 0 | 1 |
| *GmCYP51* | 2 | 7 | 6 | 1 | 0 |
| *GmCYP52* | 1 | 11 | 10 | 0 | 0 |
| *GmCYP53* | 1 | 7 | 6 | 0 | 0 |
| *GmCYP54* | 4 | 13 | 12 | 1 | 0 |
| *GmCYP55* | 1 | 2 | 1 | 0 | 0 |
| *GmCYP56* | 1 | 14 | 13 | 0 | 0 |
| *GmCYP57* | 1 | 7 | 6 | 1 | 0 |
| *GmCYP58* | 3 | 2 | 1 | 1 | 0 |
| *GmCYP59* | 1 | 14 | 13 | 0 | 0 |
| *GmCYP60* | 1 | 7 | 6 | 0 | 0 |
| *GmCYP61* | 1 | 7 | 6 | 1 | 0 |
| *GmCYP62* | 4 | 8 | 7 | 0 | 0 |

CDS, protein-coding sequence; UTR, untranslated region
